# Leukocyte DNA as Surrogate for the Evaluation of Imprinted Loci Methylation in Mammary Tissue DNA

**DOI:** 10.1371/journal.pone.0055896

**Published:** 2013-02-07

**Authors:** Ludovic Barault, Rachel E. Ellsworth, Holly R. Harris, Allyson L. Valente, Craig D. Shriver, Karin B. Michels

**Affiliations:** 1 Obstetrics and Gynecology Epidemiology Center, Department of Obstetrics, Gynecology and Reproductive Biology, Brigham and Women's Hospital, Harvard Medical School, Boston, Massachusetts, United States of America; 2 Clinical Breast Care Project, Henry M. Jackson Foundation for the Advancement of Military Medicine, Windber, Pennsylvania, United States of America; 3 Clinical Breast Care Project, Windber Research Institute, Windber, Pennsylvania, United States of America; 4 Clinical Breast Care Project, Walter Reed Army Medical Center, Washington, District of Columbia, United States of America; 5 Department of Epidemiology, Harvard School of Public Health, Boston, Massachusetts, United States of America; University of Bonn, Institut of Experimental Hematology and Transfusion Medicine, Germany

## Abstract

There is growing interest in identifying surrogate tissues to identify epimutations in cancer patients since primary target tissues are often difficult to obtain. Methylation patterns at imprinted loci are established during gametogenesis and post fertilization and their alterations have been associated with elevated risk of cancer. Methylation at several imprinted differentially methylated regions (*GRB10 ICR*, *H19 ICR*, *KvDMR*, *SNRPN/SNURF ICR*, *IGF2 DMR0*, and *IGF2 DMR2*) were analyzed in DNA from leukocytes and mammary tissue (normal, benign diseases, or malignant tumors) from 87 women with and without breast cancer (average age of cancer patients: 53; range: 31–77). Correlations between genomic variants and DNA methylation at the studied loci could not be assessed, making it impossible to exclude such effects. Methylation levels observed in leukocyte and mammary tissue DNA were close to the 50% expected for monoallellic methylation. While no correlation was observed between leukocyte and mammary tissue DNA methylation for most of the analyzed imprinted genes, Spearman's correlations were statistically significant for *IGF2 DMR0* and *IGF2 DMR2,* although absolute methylation levels differed. Leukocyte DNA methylation levels of selected imprinted genes may therefore serve as surrogate markers of DNA methylation in cancer tissue.

## Introduction

The epigenetic code allows cell function and phenotype to vary without alteration of the DNA sequence. There is increasing evidence that epigenetic mechanisms are involved in disease processes, making epigenetic marks candidates for risk or early detection markers. The tissue-specificity of epimutations represents a challenge, however, since many target tissues cannot be routinely collected in phenotypically healthy individuals. Surrogate tissues, such as peripheral blood, buccal cells, saliva and urine, would provide alternatives should they carry some of the same epimutations as the target tissue.

Imprinted genes are monoallelically expressed according to their parental origin [1], and many of them are candidates for oncogenes or tumor suppressor genes [2]. Their expression is largely controlled by specific DNA regions defined by distinct methylation patterns. These regions are called imprinting control regions (ICRs) if established in the germline, or somatic differentially methylated regions (sDMRs) if established post-fertilization [3]. Aberrant methylation at these sites is implicated in a variety of childhood syndromes, such as the Beckwith-Wiedemann, Prader-Willi, Angelman and Silver-Russell syndromes [4], [5], [6]. Altered methylation of DMRs has also been found in cancer cell lines and various primary tumor tissues [7], [8], [9], and might precede carcinogenesis.

Matched samples of blood and mammary tissue from women with and without invasive breast cancer were used to evaluate the domains of regulation of imprinted genes, in order to understand their variability and evaluate their potential as biomarkers of cancer. The imprinted genes studied were: *GRB10 ICR*, *IGF2 DMR0* and *IGF2 DMR2,* which are implicated in the insulin-like growth factor signaling pathway that is often deregulated in breast cancer [10], [11]; *H19 ICR,* which is a potential tumor suppressor gene [12]; *KvDMR* [13] and *SNRPN/SNURF ICR* [14], which are known to be altered in different human cancers.

## Materials and Methods

### Study population

The Clinical Breast Care Project (CBCP) is a clinical and research program that began enrolling patients in 2001. The primary clinical arm of the CBCP is the Clinical Breast Care Center at Walter Reed Army Medical Center. Additional recruitment centers include the Joyce Murtha Breast Care Center (Windber, PA, USA) and the Anne Arundel Medical Center (Annapolis, MD, USA).

For inclusion in the CBCP, patients were required to meet the following criteria: 1) over the age of 18 years; 2) mentally competent and willing to provide informed consent; and 3) presenting to the breast centers with evidence of possible breast disease, for routine screening mammograms or elective reductive mammoplasty. Patients were provided with layered consent forms that included permission to gather samples of breast and metastatic tissues as well as blood, and a description of primary research uses of the samples. Once informed consent was granted, the core questionnaire, with over 500 fields of information, was completed with the help of a nurse case manager, and an extensive pathology checklist was completed by a dedicated breast pathologist.

### Sample collection

For this study, clinical data, buffy coat and mammary tissue DNA were obtained from 13 women with non-proliferative benign conditions (no abnormalities, fat necrosis, or post-surgical changes), 39 patients with proliferative benign diseases without atypia (fibroadenoma (N = 23), fibrocystic changes (N = 10), stromal fibrosis or others (N = 6)), and 35 patients with invasive breast cancer (infiltrating ductal carcinoma (IDCA) (N = 31), mixed ductal and lobular carcinoma (N = 2), and other (N = 2)). Women with non-proliferative and proliferative benign conditions did not significantly differ in DNA methylation at these loci (Figure S1). Therefore both subgroups were collapsed into a single benign breast disease category for further analyses.

Prior to treatment, up to 20 milliliters of blood was collected into clot-activator and sodium heparin tubes for the isolation of DNA, and the samples were processed immediately. Following centrifugation, serum and/or plasma were separated from the blood cells and all materials stored at −80°C. Genomic DNA was isolated using Clotspin and Puregene DNA purification kits according to manufacturer's specifications (Qiagen, Valencia, CA, USA) and diluted to a standard concentration of 25 ng/ul. Tissue was collected from patients undergoing surgical procedures, including lumpectomy, mastectomy, or reductive mammoplasty. Within ten minutes of surgical removal, breast tissue was taken on wet ice to the pathology laboratory where a licensed pathologist or pathologist's assistant performed routine pathology analyses (gross characterization, margin status assessment and other indicators). Excess tissue (cancerous and/or benign) was frozen for downstream research purposes. Genomic DNA was isolated from the frozen tissue specimens after laser-microdissection of the region of interest using the ASLMD system (Leica Microsystems, Wetzlar, Germany) for invasive tissue, or after homogenization for benign tissue. Microdissected or homogenized specimens were incubated in proteinase K at 37°C overnight and then passed through purification columns (Millipore, Billerica, MA, USA). Blood and tissue samples were collected with approval from the Walter Reed Army Medical Center Human Use Committee and Institutional Review Board. All subjects enrolled in the CBCP voluntarily agreed to participate and gave written informed consent. Aliquots of approximately one microgram of DNA (less for tissue DNA) were provided for this study, with the remaining DNA being used for other research projects involving partners of the CBCP.

### DNA Methylation Assays

Bisulfite conversion was performed in duplicate, on two different days, using the EZ DNA Methylation Gold kit (Zymo Research, Irvine, CA, USA) according to the manufacturer's Alternative Protocol 2. Two-hundred-and-fifty ng of DNA was used for each duplicate conversion, and samples were eluted in 40 ul of elution buffer. PCR amplification of regions of interest was performed using 3 microliters of bisulfite-converted DNA and 0.2 uM of each primer with the HotstarTaq plus Master Mix (Qiagen, Valencia, CA, USA). Primer sequences and cycling conditions are available in Figure S2. The primer sequences for *IGF2 DMR0* [15], *IGF DMR2* [16], and *H19* [17] have been previously described. Each assay included a bisulfite conversion control to verify full conversion of the DNA.

Methylation was analyzed by highly quantitative bisulfite pyrosequencing [18] using the PyroMark Q24 system (Qiagen, Valencia, CA, USA). Pyrophosphates released during the incorporation of individual nucleotides into the elongating DNA strand are proportionally converted into light by a series of enzymatic reactions, and the light is then detected by a camera and used to calculate the percentage methylation at each CpG dinucleotide.

Values for each methylation assay were calculated by taking the average methylation score across six CpG dinucleotides for *GRB10 ICR* and *IGF2 DMR0*, seven CpGs for *IGF2 DMR2,* eight for the *H19 and SNRPN/SNURF ICRs*, and nine for *KvDMR*. The number of assessed CpG sites was dependent upon the neighboring sequence, and was determined by the PyroMark Assay Design 2.0 software (Qiagen, Valencia, CA, USA).

Each assay was validated by means of a series of standards of 0, 20, 40, 60, 80 and 100%-methylated DNA. The standards were created in quadruplicate from whole genome amplified DNA, representing 0% methylation, and DNA treated with CpG methyltransferase M.SssI (New England Biolabs, Ipswich, MA, USA), representing 100% methylation, which were mixed in relative proportions to create the series (Figure S2).

Batch effect was avoided by replicating experiments from two independent bisulfite treatments, with separate PCR and pyrosequencing experiments performed for each replicate.

The standard deviation for each population was calculated at each CpG site and used to define a threshold to exclude replicates with high variability, defined as a greater than two-standard-deviation difference between replicates at multiple sites. Where variability was high between replicates, repeats were performed using two new samples of bisulfite-converted DNA until satisfactory results were obtained or DNA stocks were exhausted. The failure to determine the average methylation at one or more loci was due to poor reproducibility of assays for samples of lower quality, quantity, or incomplete bisulfite conversion.

Correlation between genetic variants and DNA methylation could not be assessed due to insufficient quantities of DNA being available for some of the samples following methylation analysis.

### Statistical analyses

For each assay, the percent-methylation was calculated from the average across the mean of the two replicates. Spearman correlation coefficients between mean DNA methylation in leukocytes and mammary tissues were calculated for each locus. Missing values were excluded pairwise. All statistical significance tests were two-sided, and an alpha-level of 0.05 was used. Bonferroni adjustment was chosen due its common use and stringency.

## Results

A total of 87 women were included in this study: 35 with invasive breast cancer and 52 with benign breast diseases (39 with proliferative and 13 with non-proliferative benign breast conditions). The characteristics of the women are summarized in Table 1. Women with invasive breast cancer were older at tissue collection and more likely to be postmenopausal than those with benign breast disease. They also had a higher BMI on average, and were more likely to have a family history of breast cancer.

**Table 1:** Characteristics of the 87 women included in this study, drawn from the Clinical Breast Care Project.

	Benign N = 52	Invasive N = 35
**Age at tissue collection (years)**		
Mean	40	53
Range	[18–81]	[31–77]
**Race/Ethnicity (%)** 1		
White	32(61.5)	24 (68.6)
African American	16(30.8)	7 (20)
Other	4(7.7)	3 (8.6)
**BMI (kg/m^2^)**		
Mean	25.13	28.71
Range	[17.14–36.99]	[18.82–41.67]
**Familial cancer history**		
1^st^ degree relative	3	3
2^nd^ degree relative	18	16
**Age at 1^st^ period (years)**		
Mean	13	12
Range	[9–17]	[10–16]
**Menopausal status (N(%))** 1		
Premenopausal	40(76.9)	16(45.7)
Postmenopausal	3(5.8)	8(22.9)
Post-hysterectomy	4(7.7)	6(17.1)
Surgical menopause	3(5.8)	4(11.4)
Hormone Receptors Status		
Estrogen Receptor Positive only		20(57.1)
HER2 positive		9(25.7)
Triple negative		6(17.1)

1 Percentages may not add up to 100 due to missing values.

Methylation levels were successfully assessed in peripheral blood samples from 87 women for *GRB10 ICR*, 81 women for *H19 ICR*, 85 women for *KvDMR*, 85 women for *SNRPN/SNURF ICR*, 86 women for *IGF2 DMR0*, and 85 women for *IGF2 DMR2*. In mammary tissue, methylation levels were successfully assessed in 85 women for *GRB10 ICR*, 77 women for *H19 ICR*, 76 women for *KvDMR*, 69 women for *SNRPN/SNURF ICR*, 69 women for *IGF2 DMR0*, and 65 women for *IGF2 DMR2*.

### CpG methylation patterns at the loci

Using pyrosequencing, the percent-methylation was individually determined at multiple CpG sites across each locus. Methylation profiles were not uniform and demonstrated small inter-CpG site differences (Figure S3). In the entire study population, the average standard deviation across CpG sites for each locus were 4.7% for *GRB10 ICR*, 5.9% for *H19 ICR*, 3.8% for *KvDMR*, 4.8% for *SNRPN/SNURF ICR*, 10.2% for *IGF2 DMR0*, and 9.1% for *IGF2 DMR2*.

**Figure 1:**
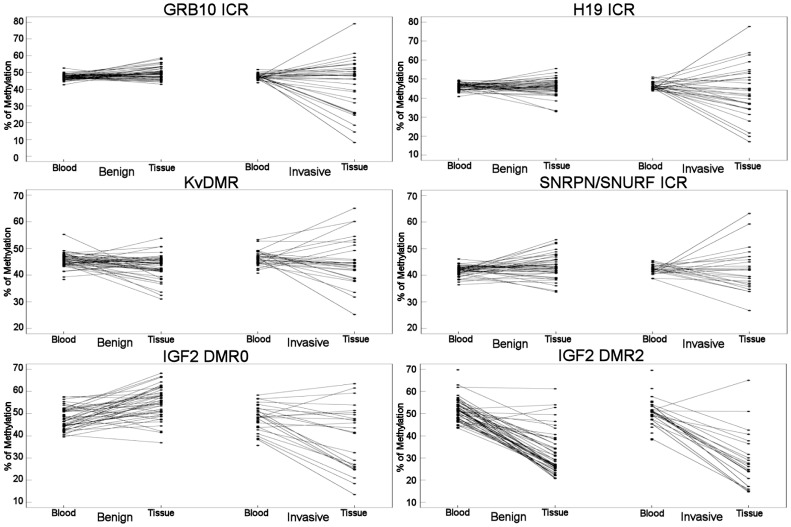
Comparisons of methylation values between blood and matching mammary tissue in different subgroups of patients. Black circles correspond to the methylation value for an individual participant.

The average methylation at each locus demonstrated little variation in women without breast cancer, with values close to the expected 50% mark (Figure S3). Only *IGF2 DMR2* showed a methylation value lower than expected in mammary tissue DNA, but this was not accompanied by a corresponding lower value in leukocyte DNA (Figure S3). Where methylation values differed from the expected 50% value, this was the result of changes across all CpG sites at the locus, rather than large singular changes. Methylation levels were more variable in mammary tissue than in leukocytes (Fig. 1). Average methylation in leukocyte DNA ranged from 36.5% to 55.3% at ICRs and from 39.5% to 69.7% at sDMRs in women free of cancer, and between 38.7% and 53.3% at ICRs and between 35.7% and 69.5% at sDMRs in women with invasive breast cancer. In mammary tissue, DNA methylation values ranged between 31.1% and 58.6% at ICRs and between 20.8% and 68.3% at sDMRs in women free of breast cancer, and between 8.3% and 79.0% at ICRs and between 13.5% and 65.5% at sDMRs in women with invasive breast cancer. Significant correlation between somatic and germinal domains within the same locus (H19/IGF2) was only observed in mammary tissues between *H19 ICR* and *IGF2 DMR0* (data not shown).

### Correlation between DNA methylation levels in leukocytes and mammary tissues

**Figure 2:**
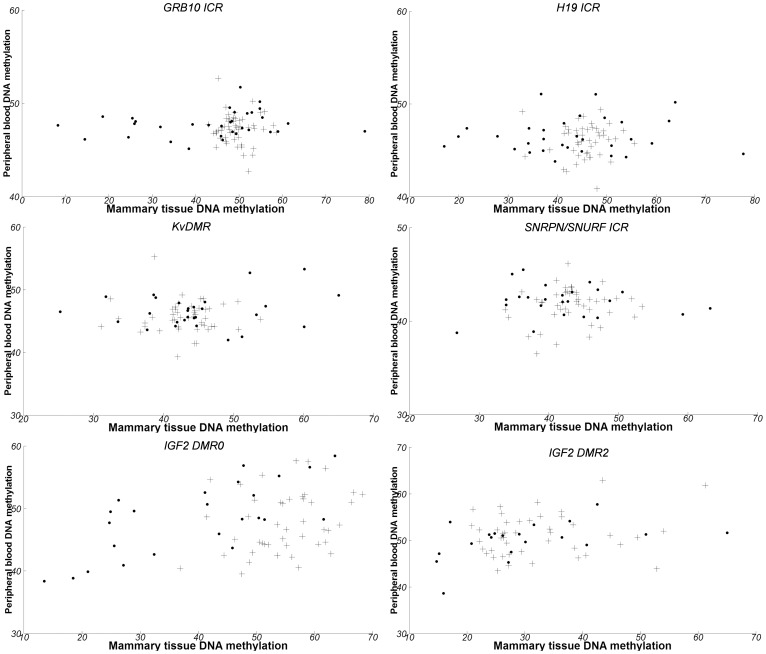
Comparisons between blood and mammary tissue DNA methylation. Axes correspond to percent-methylation observed in blood and mammary tissue, respectively. Crosses correspond to non-cancerous cases, and circles to invasive cases.

To establish whether variation of methylation in mammary tissue is reflected in leukocyte DNA, correlations between tissue and leukocyte DNA methylation were computed for each locus (Table 2, Fig. 2). Only *IGF2 DMR0* (rho = 0.65, p<0.001) and *IGF2 DMR2* (rho = 0.50, p = 0.026) displayed a significant correlation between tissue and blood in women with invasive breast cancer but not in women free of breast cancer. Only the *IGF2 DMR0* correlation remained significant after correction for multiple comparisons (Bonferroni-adjusted p = 0.0036). Stratification according to hormonal receptors status was plotted (Figure S4), but correlations could not be calculated due to the small size of subgroups.

**Table 2:** Average percent-methylation in leukocyte and mammary tissue DNA from women with benign breast disease and invasive breast cancer.

	Locus	N	Blood	Tissue	Spearman's ρ	p value of Spearman's ρ
Benign	*GRB10 ICR*	52	47.26±1.60	49.34±3.53	0.02	0.88
	*H19 ICR*	46	45.94±1.78	45.68±4.30	0.09	0.57
	*KvDMR*	47	45.50±2.61	43.13±4.42	0.15	0.35
	*SNRPN/SNURF ICR*	45	41.73±1.86	43.27±4.37	0.12	0.47
	*IGF2 DMR0*	44	47.49±4.90	54.80±6.92	0.23	0.16
	*IGF2 DMR2*	44	51.61±5.09	32.30±9.66	0.18	0.29
Invasive	*GRB10 ICR*	33	47.70±1.46	43.60±14.97	0.23	0.19
	*H19 ICR*	31	46.50±1.89	43.10±13.26	0.04	0.82
	*KvDMR*	28	46.20±2.72	45.00±8.70	0.10	0.63
	*SNRPN/SNURF ICR*	24	42.40±1.56	42.20±8.06	−0.05	0.80
	*IGF2 DMR0*	24	47.80±5.40	39.50±14.51	0.65	**6 10^−4^**
	*IGF2 DMR2*	20	51.00±5.98	30.20±12.40	0.50	**2.61 10^−2^**

N corresponds to the number of samples for which methylation values are available for both blood and mammary tissue. Values are means ± standard deviations. Spearman's ρ was calculated excluding pairs with unknown values.

## Discussion

This study was focused on imprinted loci that are under the control of differentially methylated domains [3] and have been demonstrated to be frequently altered in cancer [19], [20], but which are expected to be conserved in healthy individuals. The regulatory domains of these genes were investigated to determine their variability and to evaluate their potential as biomarkers for cancer in both blood and mammary tissue. Low interindividual variation of methylation was observed at imprinted loci in mammary tissue from women free of cancer, but considerable variation was observed in women with invasive cancer. No significant correlation was observed between the levels of DNA methylation at the analyzed loci in mammary tissue and peripheral blood in women with benign disease, but significant correlations were identified in cancer patients for *IGF2 DMR0* and *IGF2 DMR2.*

Methylation profiles at imprinted loci were relatively uniform across CpG sites but displayed inter-CpG variation similar to those observed by Ito at al. [15]. This observation suggests that individual CpG dinucleotides do not adequately describe methylation at a gene locus and highlights the need for techniques other than those based upon the use of restriction enzymes or single nucleotide extension in order for more than one CpG site to be analyzed per locus. These inter-CpG variations were higher in the somatic DMRs (around 9%) compared to the germline ICRs which demonstrated low variation (around 5%) regardless of the parental origin of methylation. These results differ from the observations of Woodfine *et al*. [16], who reported higher inter-CpG variation in paternally methylated DMRs (*e.g*. *H19 ICR*, *IGF2 DMRs*) than those of maternal origin. As the same CpG dinucleotides at the *H19 ICR* locus were analyzed in this study and that of Woodfine and colleagues [16], but using different primers, this discrepancy may be assay-related. Alternatively, the small number of samples in the previous study for inter-CpG variability assessment (N = 8) may have led to an overestimation of the inter-CpG variation. The average methylation level at each CpG site across the *IGF2 DMR0* locus in breast tissue was similar to that reported by Ito and colleagues [15].

There was significant correlation between methylation at the *IGF2 DMR0* and *H19 ICR* loci in mammary tissues. This is in contrast to findings elsewhere in ovarian carcinomas [21] and Wilms' tumors [22], but are similar to observations with colorectal cancers [23].

With the exception of *IGF2 DMR2* in mammary tissue, the average methylation values observed in the benign subgroup for each imprinted locus were close to the expected 50% (representing monoallelic methylation), suggesting that the methylation control of these domains is well conserved. However, small but significant differences in the methylation values were observed between blood and tissue from the same individual, suggesting tissue-specificity as previously demonstrated for expression [24] and for methylation at non-imprinted loci [25].

Talens *et al.* [26] recently proposed the use of leukocyte DNA for epigenetic epidemiology studies, since these samples are most commonly available in existing biobanks. The authors highlighted the need to assess each combination of locus, tissue and disease in order to establish the potential use of leukocyte DNA as a surrogate; however, in their study Talens and colleagues only examined methylation in healthy individuals and found good correlations between DNA methylation levels in leukocytes and their tissue of interest (buccal cells) for imprinted loci (*IGF2* and *KCNQ1OT1* which corresponds to *KvDMR*) and non-imprinted genes (*IGF2R*, *CRH*, *IL10*, *LEP*, *INSIGF*, *APOC1*).

Recently, Cui *et al.* reported a significant correlation in *IGF2 DMR0* methylation between colonic mucosa and peripheral blood in healthy individuals, and altered DNA methylation in both tissues among colorectal cancer patients [8]. However, Kaaks, *et al*. did not observe an increased risk of colon cancer associated with methylation changes at *IGF2 DMR0* when using a more sensitive and quantitative technique (short oligonucleotide mass analysis, SOMA) than classical bisulfite sequencing [27]. Furthermore, Ito *et al.* observed differences in *IGF2 DMR0* methylation between colorectal tumors and adjacent normal tissue but not in peripheral blood of patients with and without colorectal cancer in an unrelated population, using a threshold of ≤35% methylation to classify hypomethylation. Due to the studies being conducted in separate populations, the correlation between methylation in tissue and blood could not be assessed [15].

Previous studies conducted elsewhere have suggested that SNPs can affect the methylation status of a DNA region, increasing the probability of inter-tissue positive correlation. Most reported associations (95%) between SNPs and DNA methylation occur with SNPs located within 149 kb of the CpG dinucleotides [28]. The IGF2 domain has been investigated for SNP/methylation correlations [29], but only one, rs2239681, has been correlated with methylation at *IGF2 DMR0*, and with a very low effect (beta value = −1.3%). This suggests that this SNP would have a low impact on the results in this study, although the availability of such data here would have increased the validity of the results by removing the bias caused by genetic variants. This hypothesis could not be assessed due to a lack of DNA, but it should be taken into account for future studies.

A small number of studies have investigated *IGF2* methylation in breast cancer. Some early studies focused on allele-specific expression of the gene, with McCann and colleagues having observed biallelic expression of *IGF2* in three of five informative breast carcinomas [30], while Yballe, *et al.* identified loss of *IGF2* imprinting in 2 of 17 breast tissues [31]. Elsewhere, Wu *et al.* reported that 9 of 12 breast cancer samples displayed biallelic expression of *IGF2,* suggesting a relaxation of imprinting [32]. Van Roozendaal *et al.* observed biallelic expression of *IGF2* in three of four primary breast tumors examined, and also in the adjacent histologically normal tissue [33], while Yun and colleagues in New Zealand observed biallelic expression in 6 of 44 breast tumors and also in 2 of 13 normal breast tissues [34]. Loss of imprinting of IGF2 has also reported in half of 47 breast cancer tissues in one study in China [35], and another study conducted in India reported biallelic expression in 3 of 10 breast tumor samples [36].

Ito and colleagues reported lower *IGF2 DMR0* methylation levels in 13 of 22 mammary tumor samples compared with histologically normal breast tissue from the same patient, with 7 tumor samples displaying less than 35% methylation [15]. However, *IGF2* methylation levels did not correspond with loss of imprinting. No association between *IGF2 DMR0* methylation and breast cancer incidence was found in prediagnostic blood samples from another cohort [15], and Ito *et al*. therefore concluded that the observed hypomethylation of *IGF2* was somatically acquired, rather than an innate epimutation.

Despite differences in the absolute methylation level between the two tissues, statistically significant correlations were observed for *IGF2 DMR0* and *IGF2 DMR2* between blood and tumor tissue among women with invasive breast cancer, indicating that the ranking of individuals with respect to their methylation level is largely preserved in peripheral blood samples from breast cancer patients, but not among women free of breast cancer. This may allow derivation of a calibration equation to predict levels of DNA methylation in the tissue based upon those in the blood, with a greater sample size and normal data distribution permitting the use of parametric statistical models. If a calibration model of adequate sensitivity and specificity can be derived, it could then be used in populations for which only peripheral blood samples are available to identify individuals with subclinical breast cancer. Whether *IGF2* may be a peripheral blood marker of breast cancer remains to be established and requires further studies with larger sample sizes. While no significant correlation was observed between methylation in mammary tissue and leukocyte DNA for the other imprinted loci studied, other epigenetic biomarkers of breast cancer may be detectable in peripheral blood.

## Supporting Information

Figure S1 **Methylation distribution according to disease subtype.** White box plots correspond to benign conditions, grey plots correspond to invasive breast cancer. 1: non-proliferative conditions; 2: proliferative fibroadenomas; 3: proliferative fibrocystic changes; 4: proliferative stromal fibrosis and other benign conditions; 5: Infiltrating Ductal Carcinoma (IDCA); 6: Other invasive breast carcinomas. Bold lines represent the medians, the boxes denote the 25th and 75th percentiles, the T bars correspond to the minimum and maximum. Circles represent outliers.
(DOC)

Figure S2 A) Primer sequences and PCR conditions of the different pyrosequencing assays. B) Methylation scales performed using mix of whole genome amplified (WGA) DNA (expected 0%) and MssI treated WGA DNA (expected 100%). Each point of the scale was performed in quadruplicate. The line is the average linear regression with its coefficient of determination (R^2^).
(DOC)

Figure S3 **Average methylation profile by CpG site for each gene locus examined. Leukocyte (square and green line) and mammary tissue (pink line and diamonds) DNA from women free of breast cancer.** Leukocyte (X and blue line) and mammary tissue (plus and yellow line) DNA from women with invasive breast cancer.
(DOC)

Figure S4 **Comparisons of methylation values between blood and matching mammary tissue in different subgroups of patients stratified on hormonal receptors status.** Each black dot corresponds to the methylation value of an individual participant.
(DOC)
