# Supplementary material for: Leukocyte DNA as Surrogate for the Evaluation of Imprinted Loci Methylation in Mammary Tissue DNA
Source: PLoS One. 2013 Feb 7;8(2):e55896. doi: 10.1371/journal.pone.0055896 (PMC3567003; doi:10.1371/journal.pone.0055896)
Supplement: Figure S1 — Methylation distribution according to disease subtype. White box plots correspond to benign conditions, grey plots correspond to invasive breast cancer. 1: non-proliferative conditions; 2: proliferative fibroadenomas; 3: proliferative fibrocystic changes; 4: proliferative stromal fibrosis and other benign conditions; 5: Infiltrating Ductal Carcinoma (IDCA); 6: Other invasive breast carcinomas. Bold lines represent the medians, the boxes denote the 25th and 75th percentiles, the T bars correspond to the minimum and maximum. Circles represent outliers. (DOC) [file pone.0055896.s001.doc]

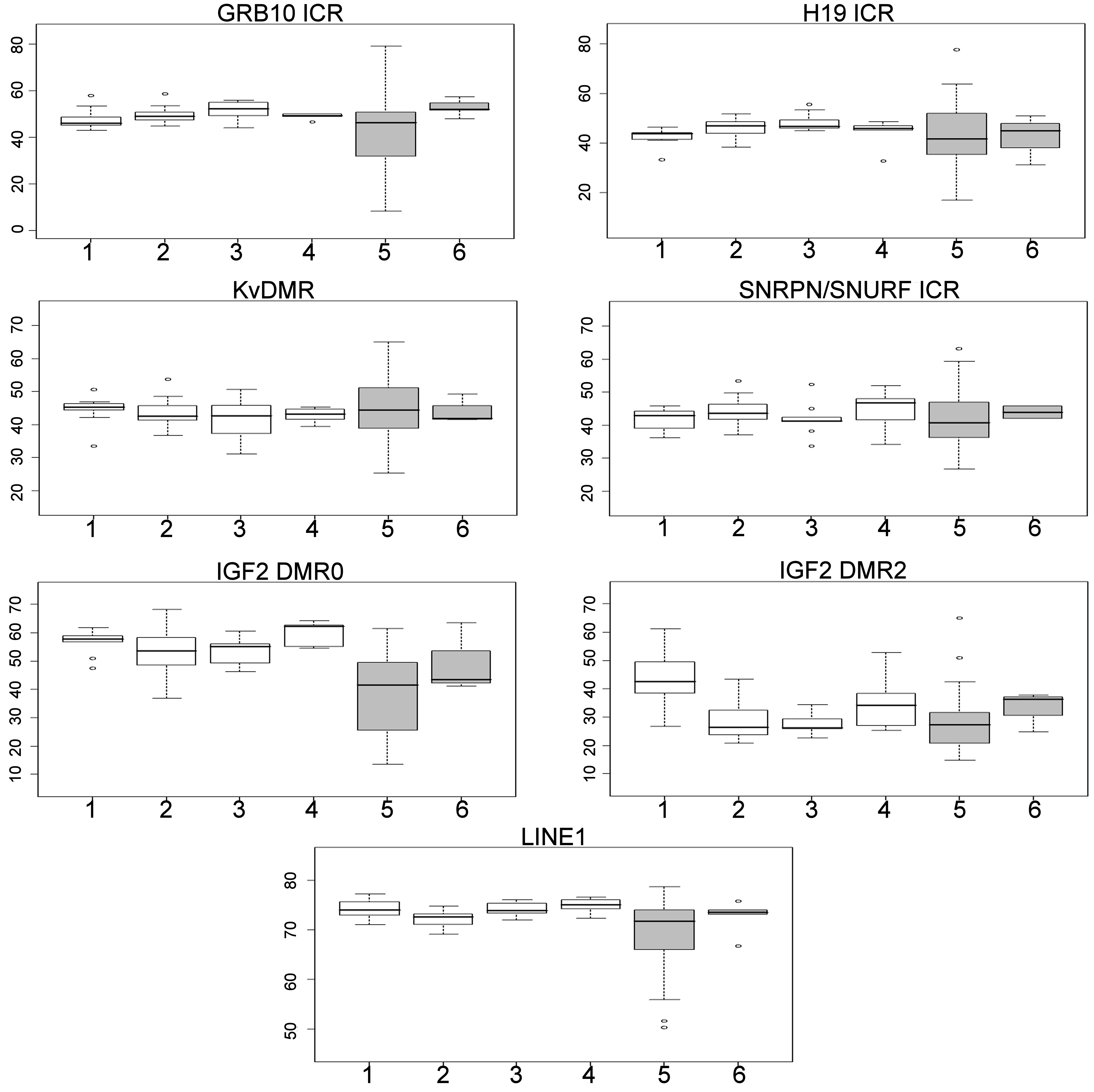


**Figure S1**: Methylation distribution according to disease subtype. White box plots correspond to benign conditions, grey plots correspond to invasive breast cancer. 1: non-proliferative conditions; 2: proliferative fibroadenomas; 3: proliferative fibrocystic changes; 4: proliferative stromal fibrosis and other benign conditions; 5: Infiltrating Ductal Carcinoma (IDCA); 6: Other invasive breast carcinomas. Bold lines represent the medians, the boxes denote the 25th and 75th percentiles, the T bars correspond to the minimum and maximum. Circles represent outliers.
