# Supplementary material for: Leukocyte DNA as Surrogate for the Evaluation of Imprinted Loci Methylation in Mammary Tissue DNA
Source: PLoS One. 2013 Feb 7;8(2):e55896. doi: 10.1371/journal.pone.0055896 (PMC3567003; doi:10.1371/journal.pone.0055896)
Supplement: Figure S2 — A) Primer sequences and PCR conditions of the different pyrosequencing assays. B) Methylation scales performed using mix of whole genome amplified (WGA) DNA (expected 0%) and MssI treated WGA DNA (expected 100%). Each point of the scale was performed in quadruplicate. The line is the average linear regression with its coefficient of determination (R2). (DOC) [file pone.0055896.s002.doc]

A)

| Primers | Sequence before bisulfite treatment | 5'-3' sequence | **UCSC Human Feb. 2009 (GRCh37/hg19) Build** | Tm  °C | Reference |
| --- | --- | --- | --- | --- | --- |
| GRB10 ICR forward | GAGGAGGCAGTGGAGGGAACAAG | GAGGAGGTAGTGGAGGGAATAAG | chr7:50,850,589-50,850,718 | 58 |  |
| GRB10 ICR reverse | ATCCCAGGACCAAACCCATGT | ATCCCAAAACCAAACCCATAT |  |  |
| GRB10 ICR sequencing | TGGGCAGCAGAGGGC | TGGGTAGTAGAGGGT |  |  |  |
| Sequence to analyze | CCCCGCCGGGCGTTGGGGCGTGGCCGCGTCACATGG  GTTTGGTCCTGGGATTC | TTTYGTYGGGYGTTGGGGYGTGGTYGYGTTATATGG  GTTTGGTTTTGGGATTT | chr7:50,850,587-50,850,639 |  |  |
|  |  |  |  |  |  |
| H19 ICR forward | TGAGTGTCCTATTCCCAGATGACCCC | TGAGTGTTTTATTTTTAGATGATTTT | chr11:2,021,077-2,021,201 | 56 | (G*uo et a*l. 2008) |
| H19 ICR reverse | GCAGTGCAGGCTCACACATCACAGC | ACAATACAAACTCACACATCACAAC |  |  |
| H19 ICR sequencing | TGAGTGTCCTATTCCCAGATGACCCC | TGAGTGTTTTATTTTTAGATGATTTT |  |  |  |
| Sequence to analyze | CGTGAACCCTGCGACGCGTGGCTTGGGTGACCCGGGA  CGTTTCCACGGGCGAACCCCAATTGGGGCGGGCT | YGTGAATTTTGYGAYGYGTGGTTTGGGTGATTYGGG  AYGTTTTTAYGGGYGAATTTTA | chr11:2,021,103-2,021,173 |  |  |
|  |  |  |  |  |  |
| SNRPN/SNURF ICR forward | GGGAGGGAGCTGGGACCCCTG | GGGAGGGAGTTGGGATTTTTG | chr15:25,200,037-25,200,201 | 60 |  |
| SNRPN/SNURF ICR reverse | AAGCCACCGGCACAGCTGACCTTGCCC | AAACCACCCACACAACTAACCTTACCC |  |  |
| SNRPN/SNURF ICR sequencing | AGCTGGGACCCCTGCACTG | AGTTGGGATTTTTGTATTG |  |  |  |
| Sequence to analyze | CGGCAAACAAGCACGCCTGCGCGGCCGCAGAGGCAGG  CTGGCGCGCATGCTCAGGCGGGGATG | YGGTAAATAAGTAYGTTTGYGYGGTYGTAGAGGTAG  GTTGGYGYGTATGTTTAGGYGGGGATG | chr15:25,200,036-25,200,098 |  |  |
|  |  |  |  |  |  |
| KvDMR forward | ACCTCAGGGGGTGAGTGGCA | ATTTTAGGGGGTGAGTGGTA | chr11:2,721,910-2,722,119 | 56 |  |
| KvDMR reverse | GCTTTTGTGACCCAGGCTTTTGTCCC | ACTTTTATAACCCAAACTTTTATCCC |  |  |
| KvDMR sequencing | AGGCCACCCACCTGGCAAAGG | AGGTTATTTATTTGGTAAAGG |  |  |  |
| Sequence to analyze | GCAGCGCCGAGGGCGCCCCGCGCCTGCCAGCGCCCGG  CCGGGCCCGCCCTGCCCACCTCCCATCCCCCATC | GTAGYGTYGAGGGYGTTTYGYGTTTGTTAGYGTTYG  GTYGGGTTYGTTTTGTTTATTTTTTATTTTTTATT | chr11:2,721,948-2,722,018 |  |  |
|  |  |  |  |  |  |
| IGF2 DMR0 forward | TGAGGATGGGCTTCTGCCTGGCAC | TGAGGATGGGTTTTTGTTTGGTAT | chr11:2,169,328-2,169,582 | 56 | (I*to et a*l. 2008) |
| IGF2 DMR0 reverse | TCCTCGATCCACCCAGGGTGGTGT | TCCTCGATCCACCCAAAATAATAT |  |  |
| IGF2 DMR0 sequencing 1 | GGGGTGGAGGGTGCA | GGGGTGGAGGGTGTA |  |  |  |
| Sequence to analyze 1 | CACGAATGGCCCGCCTTGAGGGGTCATGGCACGG | TAYGAATGGTTYGTTTTGAGGGGTTATGGTAYGG | chr11:2,169,369-2,169,402 |  |  |
| IGF2 DMR0 sequencing 2 | AAAAGCCACTGGACACACAGC | AAAAGTTATTGGATATATAGT |  |  |  |
| Sequence to analyze 2 | TCTGCTTGACGAGGCCAGTGAGGGACGGCGT | TTTGTTTGAYGAGGTTAGTGAGGGAYGGYGT | chr11:2,169,490-2,169,520 |  |  |
|  |  |  |  |  |  |
| IGF2 DMR2 forward | GGTCAGGAGGAGGCTGCAGG | GGTTAGGAGGAGGTTGTAGG | chr11:2,154,160-2,154,457 | 58 | (Woodfi*ne et a*l. 2011) |
| IGF2 DMR2 reverse | CCAGGACAACTTCCCCAGATAC | CCAAAACAACTTCCCCAAATAC |  |  |
| IGF2 DMR2 sequencing | GGGTGGGTAGAGCAA | GGGTGGGTAGAGTAA |  |  |  |
| Sequence to analyze | TCAGGGGACGGTGACGTTTGGCCTCCCTGAACGCCTC  GAGCTCCTTGGCGAGCACGTGACCCCGGCGGGCACGC  AGGAGGGCAGGCAGGCCCCTG | TTAGGGGAYGGTGAYGTTTGGTTTTTTTGAAYGTTT  YGAGTTTTTTGGYGAGTAYGTGATTTYGGYGGGTAY  GTAGGAGGGTAGGTAGGTTTTTG | chr11:2,154,285-2,154,379 |  |  |

B)

**Figure S2**: A) Primer sequences and PCR conditions of the different pyrosequencing assays. B) Methylation scales performed using mix of whole genome amplified (WGA) DNA (expected 0%) and MssI treated WGA DNA (expected 100%). Each point of the scale was performed in quadruplicate. The line is the average linear regression with its coefficient of determination (R²).
