# Supplementary material for: Leukocyte DNA as Surrogate for the Evaluation of Imprinted Loci Methylation in Mammary Tissue DNA
Source: PLoS One. 2013 Feb 7;8(2):e55896. doi: 10.1371/journal.pone.0055896 (PMC3567003; doi:10.1371/journal.pone.0055896)
Supplement: Figure S3 — Average methylation profile by CpG site for each gene locus examined. Leukocyte (square and green line) and mammary tissue (pink line and diamonds) DNA from women free of breast cancer. Leukocyte (X and blue line) and mammary tissue (plus and yellow line) DNA from women with invasive breast cancer. (DOC) [file pone.0055896.s003.doc]

**Figure S3**: Average methylation profile by CpG site for each gene locus examined. Leukocyte (square and green line) and mammary tissue (pink line and diamonds) DNA from women free of breast cancer. Leukocyte (X and blue line) and mammary tissue (plus and yellow line) DNA from women with invasive breast cancer.
