# Supplementary material for: Leukocyte DNA as Surrogate for the Evaluation of Imprinted Loci Methylation in Mammary Tissue DNA
Source: PLoS One. 2013 Feb 7;8(2):e55896. doi: 10.1371/journal.pone.0055896 (PMC3567003; doi:10.1371/journal.pone.0055896)
Supplement: Figure S4 — Comparisons of methylation values between blood and matching mammary tissue in different subgroups of patients stratified on hormonal receptors status. Each black dot corresponds to the methylation value of an individual participant. (DOC) [file pone.0055896.s004.doc]

**Figure S4**: Comparisons of methylation values between blood and matching mammary tissue in different subgroups of patients stratified on hormonal receptors status. Each black dot corresponds to the methylation value of an individual participant.
